# Factors influencing unintended pregnancy and abortion among unmarried young people in Nigeria: a scoping review

**DOI:** 10.1186/s12889-024-19005-8

**Published:** 2024-06-04

**Authors:** Love Bukola Ayamolowo, Sunday Joseph Ayamolowo, Dorcas Oluwatola Adelakun, Bukola Abimbola Adesoji

**Affiliations:** Department of Nursing Science, Obafemi Awolowo University, Ile-Ife, Nigeria

**Keywords:** Unintended Pregnancy, Abortion, Unmarried, Adolescents, Nigeria

## Abstract

**Background:**

Unintended pregnancies and abortions among unmarried adolescents in Nigeria are outcomes of the interplay of multifaceted factors. Abortion, a global public health and social issue, impacts both developed and developing countries. This scoping review explored the literature and mapped the risk factors for unintended pregnancies and abortions among unmarried female adolescents in Nigeria.

**Methods:**

A scoping literature search was conducted across databases, including PubMed, Science Direct, Web of Science, EBSCOhost, JSTOR, African Index Medicus, and Scopus. Inclusion criteria encompassed peer-reviewed articles and reports in English, focusing on unmarried female adolescents. The range of interest included the past incidents of having sex, unintended pregnancies, contraceptive use, and abortions among this demographic. Studies categorized as grey literature were excluded to ensure the reliability and validity of the synthesized information.

**Results:**

A total of 560 articles, 553 identified through databases and 7 through hand search, were subjected to a comprehensive full-text review, resulting in the inclusion of 22 studies that met the criteria for the final review. The scoping review shed light on the past incidents of having sex, unintended pregnancies, contraceptive use, and abortions among unmarried adolescents in Nigeria. The range of incidence for having sex varied from 57.2% to 82.7%, with the prevalence of unintended pregnancies ranging from 23.4% to 92.7%. Contraceptive use was notably low, with 21.5% reporting low usage, contributing to the high incidence of abortions, ranging from 20.2% to 51.0%. Factors influencing unintended pregnancies included a lack of awareness of modern contraceptives and limited access to sexual and reproductive health information. For induced abortions, factors such as the impact on educational career, childbearing outside wedlock and fear of expulsion from school were identified.

**Conclusion:**

This scoping review, through a systematic examination of existing literature, contributes to a more robust understanding of the factors influencing unintended pregnancies and abortions among unmarried adolescents in Nigeria. The findings inform future research directions and guide the development of targeted interventions to improve reproductive health outcomes for this vulnerable population.

**Supplementary Information:**

The online version contains supplementary material available at 10.1186/s12889-024-19005-8.

## Introduction

The World Health Organization and the United Nations defined adolescents as those between ages 10–19 years, youth as 15-24 years and young people as 10-24 years [1, 2] with age 15–24 accounting for about 40% of the population in Nigeria [3]. Nigeria, the most populous country in Africa, had a projected population of 216,783,381 in 2022, comprising 108,350,410 males and 108,432,971 females [4]. Nigeria is one of the less developed countries in the world with a very high population [5]. Over 30 million Nigerians are between the ages of 10–19 years, and nearly one-third of Nigeria’s total population is between the ages of 10–24 years [6]. Additionally, 63% of the population is aged 24 years or younger, with females making up an estimated 49.3% of the population [7]. Targeting individuals aged 10–24 years aligns with the critical period of adolescent growth and addresses unique reproductive health needs, informing targeted interventions aimed at supporting adolescent reproductive empowerment, promoting contraceptive use, and delaying the age of sexual debut. During this crucial period, which encompasses significant milestones such as sexual initiation, marriage, and sometimes parenthood initiation, the intricate interplay of factors affecting unintended pregnancies and abortions among unmarried adolescents is shaped [8].

Adolescent pregnancy, often unintended, defined as pregnancies in females aged 10 to 19, is a multifaceted issue with significant consequences. Low- and middle-income countries, including Nigeria, bear a substantial burden of adolescent pregnancies, with millions of pregnancies occurring among females under the age of 19 [9, 10] posing complex challenges to individuals, families, and society at large [11, 12]. For instance, data from the 2018 Nigeria Demographic and Health Survey shows that 21% of females aged 15–19 and 52% aged 20–24 have had their sexual debut in Nigeria. Additionally, approximately 3% of females aged 15–19 and 36% aged 20–24 are married or in a union [13]. However, contraceptive use among adolescents aged 15–19 is low, with only 11% of females currently using any method of contraception. In contrast, contraceptive use is higher among youth aged 20–24, with around 22% of females currently using contraception. This highlights a notable difference in contraceptive utilization between adolescents and youth in Nigeria, with higher rates observed among the older age group.

An unintended pregnancy refers to a pregnancy that is unwanted or occurs earlier than desired, happening when no children are wanted at that time [14]. Such pregnancies often occur among adolescent girls and their partners when family planning methods are not used or are used incorrectly [15]. Additionally, unintended pregnancies can result from coerced sex, which is frequently associated with a higher risk of unsafe abortions [15]. Early sexual onset has been observed to be associated with unintended pregnancies as these teenagers are more exposed to risks over a longer time, have more sexual partners, and engage in higher sexual risk behaviours [16]. Low contraceptive use contributes to a higher incidence of unintended pregnancies among adolescents [17], which in turn increases the likelihood of resorting to abortion as a means of managing unintended pregnancies. The prevalence of unintended pregnancy among adolescents in Nigeria reflects a complex interplay of factors such as early onset of menarche, early initiation of sexual activity in about 20% of the adolescents [18], early marriage, ineffective use of contraception, and limited access to comprehensive sexual education [19–22].

The prevalence rate of abortions in Nigeria is difficult to ascertain due to severe restrictions on abortion [23], as it is only permitted when necessary to save the woman's life [24–26]. Due to the legal restrictions on abortion in Nigeria, access is particularly challenging for adolescents, who often face barriers in obtaining sexual and reproductive health services. Consequently, adolescents frequently resort to unsafe abortions, posing significant health risks, increasing maternal mortality rates, and contributing to higher rates of sexually transmitted diseases [27–29] as they are exposed to more sexual intercourse and sex without a condom or any form of protection [30].

Unmarried adolescents in Nigeria, lacking support and resources, face heightened vulnerability to unintended pregnancies and abortions [31, 32]. This vulnerability is exacerbated by societal stigma surrounding premarital sexual activity, limiting their access to contraception and reproductive health education [31]. The stigma, which may carry legal and social consequences, exacerbates their challenges. Understanding their experiences is crucial for developing effective interventions and policies tailored to their reproductive health needs such as comprehensive sexual education, accessible reproductive health services, contraception availability, community outreach, and policy enhancements. With Nigeria's projected population growth, empirical data are essential for national planning for adolescent health. Therefore, this scoping review aims to explore the prevalence and associated factors of unintended pregnancies and adolescent abortions among unmarried adolescents in Nigeria. The socio-ecological model was used as a guiding framework to report factors influencing unintended pregnancy and abortion [16]. This model provides a natural theoretical framework to study and address the multiple level of influence on adolescents unintended pregnancy and induced abortion on different levels which are the individual, interpersonal, organizational and community level. Empirical findings guided by this model will help to identify and suggest strategies for reducing unintended pregnancy and induced abortion among unmarried adolescent and young people in Nigeria. It seeks to provide nuanced insights into sexual health issues among adolescents, informing interventions, policies, and strategies to address the pressing issue of unintended pregnancies.

## Materials and method

This scoping review seeks to provide nuanced insights into the multifaceted factors that contribute to unintended pregnancies and abortions among unmarried adolescents in Nigeria. Arksey & O’Malley [33] ‘York methodology’ was adopted in conducting this scoping review. This scoping review seeks to provide a broad overview of the available literature, including both older and newer studies in Nigeria. Scoping reviews can capture the breadth and evolution of research on the topic over time, ensuring a comprehensive understanding of the existing literature landscape. The review adhered to the Preferred Reporting Items for Systematic Review and Meta-Analysis (PRISMA) extension for scoping reviews checklist [34, 35].

2.1. Research question.

The research question guiding this scoping review was: What are the factors associated with unintended pregnancy and abortion among unmarried female adolescents in Nigeria?

### Identification of relevant studies

This search was conducted in November 2023 for relevant articles published in English in seven electronic databases (PubMed, Science Direct, Web of Science, EBSCOHOST, JSTOR, African Index Medicus, and Scopus) using the terms shown in Appendix A. Supplementary searches were manually performed on the reference compilations of potentially pertinent publications.

### Selection of studies for review

Identified studies were downloaded into Endnote, imported into Rayyan, and duplicates were removed. Rayyan, an automation tool, was utilized to enhance the validity of the selection process for inclusion in reviews. It facilitated the easy importation of references, enabled collaboration among researchers, and tracked screening decisions effectively. Title and abstract screening were performed independently by two researchers (LBA and SJA) using pre-defined inclusion and exclusion criteria. Articles were selected solely when there was concurrence among the two. Any uncertainties concerning the discrepancies in the eligibility of publications were resolved through mutual agreement with a third researcher (DOA). A discussion was then held, and consensus reached between the three researchers on the eligibility status of the publication. Following this, the researchers conducted individual assessments of the complete texts of the remaining articles, and supplementary searches were manually performed on the reference compilations of potentially pertinent publications.

### Inclusion criteria

Articles with reports on the factors associated with unintended pregnancy and abortion among unmarried adolescents in Nigeria within ages 10–24, published in English, and peer-reviewed were included in the review. All articles included had to have undergone peer review.

### Exclusion criteria

Excluded from consideration were articles involving participants outside of Nigeria. Additionally, articles with inaccessible full lengths, commentaries on studies, and letters to the editor were omitted. Narrative reviews that did not specifically address factors associated with unintended pregnancy and abortion among unmarried adolescents in Nigeria were also excluded. Furthermore, grey literature was not included in the analysis.

### Data charting process

A data-charting form for extracting pertinent variables was developed by the authors, who then individually carried out data extraction for each study included in the review. Two distinct authors, uninformed about each other's findings, charted the data, ensuring precision in data extraction. The authors recorded variables associated with the aim of the scoping review, such as authors and year of publication of the study, study location, study objective, study design, methodological approach for data collection, and study outcome.

### Data analysis

The extracted data from the selected studies underwent thematic analysis to reveal recurring themes, patterns, or trends within the literature. These emerging themes closely corresponded with essential concepts, issues, or findings within the review’s scope. Subsequently, the results from these studies were synthesized narratively, structured around the identified themes or key concepts, and highlighting gaps in the literature. Furthermore, the key findings were compared to determine the prevalence of contraceptive use, sexual intercourse, unintended pregnancy and abortion among adolescents in Nigeria. Subsequently, we summarized the factors responsible for the occurrence of unintended pregnancies and abortions among unmarried adolescents in Nigeria as outlined in the extracted articles using socioecological model. The data were categorized into common groups and presented in a table. The findings were then assessed in relation to the research question and objectives using a narrative summary as tabulated results. This model was adopted from a previous study [36, 37]. By considering multiple levels of influence, ecological models provide a comprehensive framework for understanding health behaviors, social dynamics, and environmental processes.

## Results

**Fig. 1:**
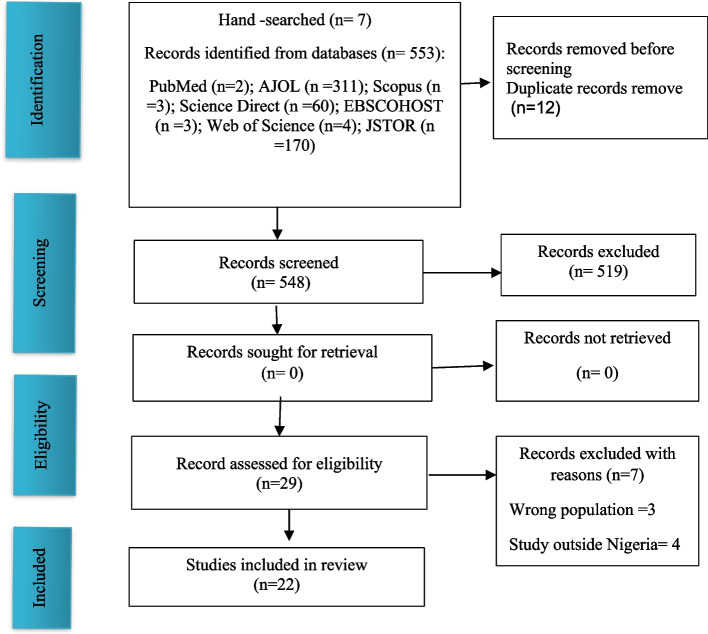
PRISMA screening process

The search yielded 560 articles, reduced to 543 after removing duplicates. Title and abstract screening identified 29 articles meeting eligibility criteria for a full-text review. After a meticulous examination, six articles were excluded due to irrelevant data or non-specificity to Nigeria. The final dataset comprised 22 articles aligning with inclusion criteria. Figure 1 visually outlines the systematic screening process. This rigorous selection ensures the relevance of the final articles to the scoping review's goals on factors influencing abortion among Nigerian adolescents.

### Characteristics of the selected studies

The 22 studies included in this review spanned publication years from 1981 to 2023 within the Nigerian context. Only one (4.5%) study [38] was conducted between 1980 and 1988. Notably, there were no studies conducted between 1990 and 1999. The subsequent decade, 2000–2009, contributed five studies (22.7%) to the body of literature [31, 38–41]. The majority of studies 10 (45.6%) emerged in the period 2010–2019 [42, 44, 48–56]. Subsequently, six studies 6 (27.3%) were conducted from 2020 onwards [26, 43–47].

### Geographical region in Nigeria

The 22 studies spanned five of the six geopolitical zones in Nigeria, with distribution as follows: *South-West* 30.4% (*n* = 7) [46, 47, 52, 53, 55, 57]. *South-South* 30.4% (*n* = 7) [26, 38–40, 42, 44, 48]. *South-East* 13% (*n* = 3) [31, 45, 56]. *North-West* 4.3% (*n* = 1) [54]. *North-Central* 13%(*n* = 3) [41, 49, 50], and none were conducted in the *North-East 0% (n* = *0)*. Additionally, two studies had nationwide scopes 8.6% (*n* = 2)[45, 51]. The studies conducted in South-West Nigeria covered four of the six states in the geopolitical zone: Lagos 28.5% (*n* = 2) [43, 57], Oyo 42.8% (*n* = 3) [52–54], Ogun 14.2% (*n* = 1) [55], and both Osun and Lagos 17.2% (*n* = 1) [47].

In South-South Nigeria, the studies were conducted in four of the six states in the region: Edo 42.8% (*n* = 3) [38, 39, 44], Rivers 28.5% (*n* = 2) [26, 42], and each in Akwa Ibom 14.2%(*n* = 1) [ [40] and Bayelsa 14.2% (*n* = 1) [48]. The two studies in Southeast Nigeria were in Anambra State 50% (*n* = 1) [31], and in Imo State 50% (*n* = 1) [56]. Meanwhile, the single study in North-West Nigeria was conducted in Kaduna State 100% (*n* = 1) [54]. In North-Central, two studies were conducted in Kwara 66.6% (*n* = 2) [41, 49], and one in Kogi State 33.3% (*n* = 1) [50] (Table 1).

**Table 1:** Characteristics of the selected studies

Author/year of publication	Geo-political zone Year of study	State	Study population	Sample size	Study objective	Study type Type of data collected/methods used	Study outcomes	Factors influencing unintended pregnancy and induced abortion
Abiodun & Balogun (2008). [41]	North-Central N/A	Kwara	Female students aged 15 -24	600	Evaluated the pattern of sexual behavior and contraceptive use among female students attending tertiary institutions in Ilorin	Cross-sectional Surveys & Questionnaires	Sexual intercourse:77.6%, Unintended pregnancy:67.8% Induced abortion:63.5% Contraceptive use:25.4%	*Unintended Pregnancy* Fear of side effects of contraceptives use
Abiodun-Ajayi et. al., (2022) [43]	South-West 2021	Lagos	Adolescents 13 to 19 years	750	Examined the determinants of teenage pregnancy and abortion among adolescents in Ayobo community, Lagos State	Cross-sectional Surveys & Questionnaires	Perceived level of teenage pregnancy and abortion: 69.4%	*Unintended Pregnancy* Lack of knowledge of Physical development, Poor Socio-economic status, Sex education, Social media
Abiola et al. (2016) [57]	South-West N/A	Lagos	Female senior secondary school students 10 to 24 years	210	Determined the knowledge, attitude, and practice of abortion and the factors associated with it among female students of two public senior secondary schools in Mainland Local Government Area, Lagos State	Cross-sectional Surveys & Questionnaires	Ever had an abortion: 2%	*Induced abortion* Age, Not yet ready for responsibility, Fear of discrimination and parental disapproval
Achema et al., (2015). [50]	North-Central N/A	Kogi	Secondary school teenage girls of Abejukolo, Omala Local Government area of Kogi state	300	To determine the perception of students about factors responsible for teenage pregnancy It's implication on adolescent health and education	Cross-sectional Surveys & Questionnaires	^a^N/A	*Unintended Pregnancy* Lack of parental care, Lack of self- control, Lack of sex education, Polygamy
Adeneye et al. (2017) [55]	South-West 2016	Ogun	Adolescents	1041	Factors associated with the prevalence of adolescent pregnancy	Cross sectional Surveys & Questionnaires	N/A	*Unintended Pregnancy* Child neglect, Ignorance of sex related issues, Condemned use of contraceptives, Perceived sex education inappropriate
Aderibigbe et al., (2011). [49]	North –Central 2006	Kwara	In school adolescents aged 10–21 of public secondary school in Ilorin	521	Examined the prevalence of teenage pregnancy, abortion among in school adolescents in Ilorin	Cross-sectional Surveys & Questionnaires	Sexual intercourse:28.2% Pregnancy rate Once:5.7% More than once: 33.3% Abortion was 100%	*Unintended Pregnancy* Male factor (Initiator of sex) Induced abortion: Male factor (Perpetrators of abortion)
Cadmus & Owoaje (2011) [52]	South-West N/A	Oyo	Female undergraduates of the University of Ibadan 15 to 30 years	425	Assessed the knowledge about complications and practice of abortion among female undergraduates of the University of Ibadan	Cross-Sectional Mixed methods Questionnaires and In-depth interview	Sexual intercourse: 28.7% Ever been pregnant: 24.5% Pregnancy ended in induced abortion: 93.3% :	*Induced abortion* Young age, not yet ready to bear responsibility of raising a child, Fear of future consequences
Ilika & Anthony (2004). [31]	South-East 2002	Anambra	Unmarried adolescents	136	Identified the characteristics and factors influencing unintended pregnancy among unmarried young women in a rural community in South-East, Nigeria	Cross-sectional Surveys & Questionnaires	Sexual intercourse: 75% Has had unintended pregnancy: 17% Contraceptive use:13.3%	*Unintended Pregnancy* Sex for material gain and economic reasons *Induced abortion* Fear of stigmatization, Partners negative reaction, Fear of school and job termination, forced marriage, parental disappointment, physical violence, verbal violence
Isa et al., (2012). [48]	South South 2011	Bayelsa	Teenage mothers	83	Determined socio-demographic factors associated with teenage pregnancy in Niger Delta	Retrospective Surveys & Questionnaires	Has had unintended pregnancy 85.5% Abortion:54.2% Contraceptive use: 25.5%	*Unintended pregnancy* Unemployment (Low social class), Lack of usage of contraceptives, educational status, marital status
Izugbara (2013) [51]	National N/A	36 states + FCT	Unmarried adolescent girls aged 15 to 19 years	6591	Investigated the socio-demographic risk factors for unintended pregnancy among unmarried Nigerian girls	Cross-Sectional Surveys & Questionnaires	Unintended pregnancy**:** 7.46%	*Unintended pregnancy* Older age of adolescents, Sex of household head (male headed), Age of household head, educational status, Poor household, Marital status
Kasso & Obidinnu (2022) [26]	South-South 2019	Rivers	Female undergraduate students	315	Determined the knowledge and prevalence of induced abortion among undergraduate students	Cross-Sectional Surveys & Questionnaires	Induced abortion:79.5% More than three abortions: 19.6%	*Induced abortion* Socio-demographic variables include: Age, Educational status, religion, place of residence, family status -polygamy, not yet ready to bear children responsibilities Parental disapproval
Murray et. al., (2006) [39]	South-South 2002	Edo	Young women 15 to 24 years	602	Assessed the prevalence of and factors with induced and abortion among young women in Edo State	Cross-Sectional Surveys & Questionnaires	Induced abortion: 41.0% Contraceptive use:20%	*Unintended pregnancy*: Living in urban areas, educational status, Alcohol consumption, Age of sexual partners, Sexual debunt, Ethnicity, Religion, Parental education
Ndifon et al., (2006). [49]	South-South 2004	Akwa Ibom	Female student Nurses	195	Understood the sexual behaviors, contraceptive practices and interventions adopted following contraceptive failure	Cross-sectional Surveys & Questionnaires	Sexual intercourse:65.7%, Unintended Pregnancy:33.6% Induced abortion:51.2% Use of condom:37.4%	*Unintended pregnancy* Source of sponsorship, Multiple sexual partner *Induced abortion*: Older unmarried, Sponsorship by parents, Married undergraduates, Level of education
Obiyan et al., (2023) [47]	South-West 2019	Osun and Lagos	Female street involved adolescents aged 10–19	424	Determined the correlate of unwanted pregnancy and induced abortion among Sexual intercourse female street involved adolescents	Cross-sectional Mixed method Questionnaire In depth interview	Sexual intercourse:63.7% Unintended pregnancy:23.4% Induced abortion:59.4% Use of contraceptive:17% Themes: History of (unwanted) pregnancy, history of induced abortion,	*Unintended Pregnancy* Sexual and reproductive health information, not aware of modern contraceptives, sexually active, Not using a modern contraceptive, Age, Educational status *Induced abortion* Educational status, Age, Not employed, Living alone, Doubt of paternity, poor financial status, rejection of pregnancy by a sexual partner
Oghagbon & Agbede (2020) [46]	South-South 2021	Edo	In-school female adolescents	239	Investigated the environmental factors predicting unintended pregnancies among in-school female adolescents in south-south, Nigeria	Cross sectional Surveys & Questionnaires	**N/A**	*Unintended Pregnancy* Peer influence Media influence Parent support
Okereke (2010) [56]	South-East 2009	Imo	In-school and out of school adolescents aged 10–19	540	Examined the prevalence and determinants of adolescents' unintended pregnancy and induced abortion in Owerri, Nigeria	Cross-sectional Mixed methods Questionnaire Focus’s group discussion	Sexual intercourse: 57.2%, Unintended pregnancy:31%, Abortion: 20.2%	*Unintended pregnancy* Unwillingness to buy contraceptives, Low usage of contraceptives, Concerns about the side effects of contraceptives, Cultural reasons, Lack access to SRH services, Religion *Induced abortion*: fear of parental disapproval/ Humiliation, Lack of established paternity, Hindrance to getting a husband, Fear of expulsion from school
Omu et al., (1981) [38]	South-South 1980	Edo	Adolescents girls less than 20 years	244	Determined factors responsible for adolescents induced abortion Understood the consequences of adolescents induced abortion	Retrospective Surveys & Questionnaires	Abortion**:** 28.4%	*Induced abortion* Contraceptives Education, Lack of access to contraceptives, Poor motivation
Onebunne & Bello (2019). [46]	South- West 2015	Oyo	Female undergraduate of University of Ibadan	300	Assessed the prevalence of unwanted pregnancy and induced abortion among Female undergraduate	Cross-sectional Surveys & Questionnaires	Unwanted pregnancy 92.7% Induced abortion: 51.0%	*Unintended pregnancy* Marital status *Induced abortion* Marital status
Onukwugha, et al. (2020) [45]	National 2019	Multi states	Young women 15 to 24 years	45,793	Examined the trends in and individual and contextual-level predictors of pregnancy termination among 15–24-year-old women in Nigeria	Cross-Sectional Surveys & Questionnaires	Decline in abortion from 2003: 5.8% 2013: 4.2% Then 2018: 4.9%	*Induced abortion* Educational status, Households Wealth Index, Early sexual debut, Age, Ethnicity, Religion, Place of residence
Oriji et al., (2009) [42]	South- South 2009	Rivers	Female undergraduates of University of Portharcout	500	Determined the proportion of undergraduate students who had induced abortion and Examined the contributing factors to unwanted pregnancy	Cross-sectional Surveys & Questionnaires	Sexual intercourse: 82.7% Abortion: 47.2%	*Induced abortion* Affect educational career, marital status (Do not want child outside wedlock), Uncertainty about the father of the child, use as spacing of pregnancy, Rape and incestuous sexual activity
Oyefabi et al (2016) [54]	North-West 2015	Kaduna	Undergraduate students	540	Determined the prevalence, perceptions, determinants, and consequences of induced abortion among the Kaduna State University students	Cross-Sectional Surveys & Questionnaires	Sexual intercourse: 8.38% Induced abortion: 6.7%	*Induced abortion* Age, Religion, Educational level
Salami et al., (2014). [53]	South –West 2013	Oyo	Teenagers	174	Elicited intergenerational views on the influence of unmet social need on teenage pregnancy	Cross-sectional Surveys & Questionnaires	Pregnancy incidence: 86.7%	*Unintended Pregnancy* Unmet material and financial support expected from parents, Free education, Lack of sex education and knowledge needs for signs of maturity

^a^*N/A* Not Available: The study results did not report the proportions of teenagers involved in sexual activity, experiencing unintended pregnancies, or using contraception, contrasting with findings from other studies

### The design of the selected studies

The sample sizes among the 22 studies showed considerable variation, ranging from 45 to 45,793 participants. In aggregate, these studies engaged a total of 54,298 participants. Of the 22 studies, 20 (90.9%) adopted a cross-sectional design. Another study design utilized was retrospective [38, 48]. Additionally, one study employed mixed research methods [47]. No study exclusively utilized qualitative methods. All the studies were conducted in community settings, and none were facility-based surveys (Table 1).

### Sexual health behaviors exhibited by adolescents

Table 1 presents the diverse sexual health behaviors (SHB) observed among the adolescents in the study. The assessed sexual health behaviors included the prevalence of ever having had sexual intercourse among the adolescents [31, 40–42, 47, 49, 52, 56]. The proportion of respondents reporting sexual intercourse varied, ranging from 8.38% in Kaduna State [54] to 77.6% in Lagos State [41]. Among the 22 selected studies, six reported the prevalence of contraceptive use [39–42, 47, 48], with rates ranging from 13.3% in Anambra [23] to 37.4% using condoms in Akwa Ibom [40]. Additionally, one study provided a detailed breakdown of contraceptive methods used, revealing that 21.5% reported using withdrawal, 16.6% practiced abstinence, and 5.1% utilized oral pills [40].

### Prevalence of unintended pregnancy and abortion

The prevalence of unintended pregnancies [31, 40, 41, 46–49, 53, 56] and abortions [38–42, 45–47, 49, 52, 54, 56, 57] among the respondents varied significantly across different studies (Table 1).

The prevalence of sexual behavior, unintended pregnancy and abortion observed in the review varied across geopolitical zones among adolescents (10–19), young people (10–24) or youth (20–24). Among adolescents (10 to 19 years), prevalence of early sexual debut ranged between 28.2% in kwara [49] and 63.7% in Osun and Lagos [47] while among young people (10–24 years), early sexual debut ranged from 75% in Anambra [31] and 77.6% in Kwara [41] Among the youth (20–24 years), sexual intercourse ranged from 28.7% in Oyo [52] and 82.7% in Rivers [42]. The prevalence of unintended pregnancy among 10–19 years adolescents ranged between 7.46% Nationwide [51] and 85.5% in Bayelsa [48] as well as among young people (10–24 years), from 17% in Anambra [31] to 92.7% in Oyo [46] and among the youth (20–24 years), unintended pregnancy ranged from 24.5% to 92.7% in Oyo [46, 52] and 33.6% in Akwa Ibom (49). Also, the prevalence of induced abortion ranging from 2% in Lagos [57] to 100% in Kwara [49] among 10–19 years adolescents, 4.9% Nationally (45) to 79.5% in Rivers [26] among young people (10–24 years) and 6.7% in Kaduna [54] to 93.3% in Oyo [52] among the youth (20–24 years).

### Factors influencing unintended pregnancy and abortion among adolescents

The Table 2 presents factors associated with unintended pregnancy and abortion among adolescents in Nigeria using the socio-ecological model [36, 37]. The factors identified to be associated with unintended pregnancy and abortion were categorized into the individual level, interpersonal level factor, organizational level and community-level factors. The common factors for unintended pregnancies include inadequate sex education (27.3%, [38, 43, 47, 50, 53, 55]) and contraceptive usage (31.8%, [38, 41, 45, 47, 48, 55, 56]). For abortions, age (22.7%, [40, 47, 52, 54, 57]) and fear of future consequences (18%, [31, 42, 52, 56]) were prevalent. Interpersonally, parental influence (4.5%-13.6%, [26, 39, 40, 57]) plays a role. Community factors like place of residence (9.1%-13.6%, [26, 39, 51]) are significant. The study identified various socio-ecological factors influencing unintended pregnancy and abortion among unmarried adolescents. These factors include age, pregnancy unpreparedness, educational achievement, socio-economic status, early sexual debut, multiple sexual partners, family dynamics, and community determinants. Understanding these socio-ecological factors is crucial for developing effective interventions and policies to address reproductive health issues among adolescents.

**Table 2:** Social-ecological model of the associated factors of unintended Pregnancy and abortion in the selected studies

Unintended Pregnancy		
**Associated factors**	**%**	**Articles Author’s NO**
***Individual level***		
Socio economic reasons	18	[31, 43, 51, 53]
Educational status	27.3	[2, 39, 47, 48, 51, 53]
Inadequate/ lack of sex education	27.3	[38, 43, 47, 50, 53, 55]
Multiple sexual partner	4.5	[40]
Contraceptive usage	31.8	[38, 41, 45, 47, 48, 55, 56]
Age of adolescent	18.18	[39, 43, 47, 51]
Lack of self- control	4.5	[50]
Alcohol influence	4.5	[39]
Early Sexual Debut	9.1	[39, 47]
***Interpersonal level***		
Age and sex of household head	4.5	[51]
Parental influence	4.5	[39]
Peer influence	4.5	[44]
Media influence	9.1	[43, 44]
Family type	4.5	[50]
Source of sponsorship	4.5	[40]
Marital status	13.6	[46, 48, 51]
Child abuse/neglect	9.1	[50, 55]
Age of sexual partner	4.5	[39]
Access to SRH services	13.6	[39, 47, 55]
***Organizational level***		
Religion	9.09	[39, 56]
Unemployment	4.5	[48]
***Community level***		
Culture of the society	4.5	[56]
Ethnicity	4.5	[39]
Place of residence	13.6	[26, 39, 51]
**Associated factors for abortion**		
*** Individual level***		
Age	31.8	[26, 40, 45, 47, 52, 54, 57]
Unpreparedness	13.6	[26, 45, 47, 57]
Male factor	4.5	[31]
Fear of future consequences (Hinder marriage, Affect career & Educational pursuit)	18	[31, 39, 42, 52]
Educational status	22.7	[26, 40, 45, 47, 54]
Early sexual debut	4.5	[45]
Ignorance of contraceptives	9.1	[38, 56]
Pregnancy rejection by partner	4.5	[31, 45]
***Interpersonal level***		
Paternity doubt	9.1	[42, 56]
Child spacing	4.5	[42]
Parental influence	1[3.6	[26, 40, 56]
Marital status	13.6	[40, 42, 46]
***Organizational level***		
Fear of Discrimination/ Stigmatization	13.6	[31, 56, 57]
Religion	4.5	[26]
Unemployment	4.5	[56]
Fear of expulsion from school	4.5	[39]
***Community level***		
Place of residence	9.1	[26, 45]
Forced marriage	4.5	[31]
Polygamy	4.5	[52]
Ethnicity	9.1	[45, 47]
Household wealth	4.5	[45]

## Discussion

Our review of literature identified 22 articles offering comprehensive insights into factors related to unintended pregnancy and abortion among adolescents in Nigeria, covering diverse regions. The study also identifies the epidemiological profile of adolescent sexual and reproductive practices. The prevalence of sexual activity among adolescents in Nigeria varies significantly across regions, with rates as high as 77.6% in Lagos State and low contraceptive usage, notably in Anambra. This highlights the correlation between increased sexual activity and heightened rates of unintended pregnancy. Addressing these disparities requires effective policy and strategy development. For instance, comprehensive sexual education programs in schools and communities and promoting contraceptive use through awareness campaigns could provide adolescents with accurate information about contraception and reproductive health, empowering them to make informed decisions and reduce the incidence of unintended pregnancies. However, the reliance on recruiting participants from schools and communities among the identified studies overlooks adolescents seeking healthcare in hospitals and clinics. This oversight potentially excludes pregnant adolescents and those who have had abortions and need medical care. Thus, this study highlights gaps in healthcare access and utilization among adolescents, emphasizing the importance of including them in future research and interventions for a comprehensive understanding of their sexual and reproductive health needs.

### Prevalence of adolescent pregnancy and abortion

The scoping review highlights the prevalence of unintended pregnancies, abortion, and other sexual reproductive health outcomes among adolescents in Nigeria. The reported prevalence of unintended pregnancy among adolescents varied widely, ranging from 17% in Anambra State to 92.7% in Oyo State. This finding suggests significant disparities in access to reproductive health services, contraceptive use, and sexual education across different regions of Nigeria. Policymakers and healthcare providers should prioritize regions with higher rates of unintended pregnancies, such as Oyo State. This can be achieved by implementing targeted interventions to improve access to comprehensive sexual education, contraceptives, and family planning services. The prevalence of unintended pregnancies remains significant across various countries. For example, Ayalew et al. [58]. reported prevalence rates across sub-Saharan Africa and Asia, revealing rates in India (16.9%), Nepal (22.7%), Sri Lanka (17.2%), South Asian countries (19.1%), Pakistan (38.2%), Kenya (41%), and Uganda (37%). Improving access to youth-friendly reproductive health services and affordable contraceptives is crucial for reducing unintended pregnancies among adolescents. The prevalence and circumstances surrounding induced abortions among adolescents sheds light on this sensitive aspect of reproductive health [38–42, 45–47, 49, 51, 52, 54, 56, 57]. This emphasizes the need for targeted interventions including comprehensive sexual education, and access to contraception to empower adolescents in making informed choices about their reproductive health.

### Individual factors

#### Demographic factors

The Socio-demographic factors include the age of adolescents, educational status and socioeconomic reasons.

#### Age and pregnancy unpreparedness

Adolescents, particularly younger ones, may lack the emotional maturity and cognitive understanding necessary to engage in responsible sexual behavior. This immaturity can lead to impulsive decisions and a lack of foresight regarding the potential consequences of sexual activity, including unintended pregnancy. Age and unpreparedness for unintended pregnancy significantly influence the decision to undergo abortion, as indicated by multiple studies [26, 47, 52, 56]. Young individuals who feel unready for the responsibilities of parenthood may choose abortion to avoid the emotional, financial, and social challenges of raising a child [59]. Factors such as educational goals [31, 42, 52] career aspirations [39, 52], financial instability [45, 54], and societal stigma [31, 54, 57] surrounding unplanned pregnancies further contribute to this decision. It becomes crucial to provide comprehensive support systems, including access to reproductive health education and non-judgmental healthcare services, to addressing these factors and ensuring adolescents can make informed choices about their reproductive futures***.***

#### Educational achievement

Adolescent educational level plays an important role in the prevalence of unintended pregnancy in Nigeria. Inadequate sex education [26, 39, 48, 51, 53, 56] and educational status [26, 37, 39, 43, 48, 51, 53, 56] contribute to poor decision making regarding sexual activity and contraceptive usage leading to unintended pregnancy. Studies have shown that there is a significant increase in the prevalence of unintended pregnancy among teenagers if they had no education or only primary school education [26, 39, 47, 48, 51, 53]. The study also observed a significant decrease in unintended pregnancies among adolescents with a history of school attendance [47]. This finding is consistent with research from Kenya, which suggests that adolescent girls with secondary and higher education levels are less likely to experience unintended pregnancy [60]. Conversely, limited access to higher education increases the risk of unintended pregnancy among adolescent girls [61]. Educated adolescents are empowered with comprehensive knowledge of sexual and reproductive health, enabling them to delay childbearing, use contraceptives, and understand the risks of induced abortion. Expanding sex education to vocational training centers can reach more adolescents, providing accurate information on sexuality, promoting healthy relationships, and ultimately enhancing overall health outcomes.

#### Socio-economic status

Socio-economic status (SES) significantly influences both unintended pregnancies and abortion decisions, particularly among adolescents. Studies indicate that adolescents from both middle-class and poor families are susceptible to unintended pregnancies, often driven by financial strains within their households [32, 34, 39]. Financial pressures may drive adolescents to engage in sexual activity for material gain, underscoring the significant influence of SES on reproductive health outcomes [32, 34, 39]. Yakubu et al. [62] highlighted SES as a significant contributor to adolescent pregnancy, citing poverty and financial strains as factors that can lead adolescents in Sub-Saharan African to unintended pregnancy. Interventions like vocational training and affordable higher education are proposed to alleviate financial strain and potentially lower rates of unintended pregnancies [43]. Nonetheless, adolescents from lower SES backgrounds face hurdles in accessing adequate reproductive health education and services, heightening their vulnerability to unintended pregnancies [45]. The desire to pursue schooling and enhance economic prospects emerges as significant factors influencing abortion decisions [23, 39], underscoring the interconnectedness of SES, unintended pregnancies, and abortion choices. Collaborative efforts involving governments, policymakers, and healthcare providers are essential to effectively address these complex issues. Implementing initiatives to alleviate poverty and enhance access to education and reproductive health services are imperative steps toward reducing unintended pregnancies and abortions among vulnerable populations.

### Behavioural Factors

Some of the behavioural factors influencing adolescent unintended pregnancy and unsafe abortion include early sexual debut and multiple sexual partners. Early sexual debut can increase adolescent risk of multiple sexual partners as well as unprotected sexual intercourse which can lead to unintended pregnancy and the risk of engaging in induced abortion. Similarly, teenagers who initiate sex at a younger age as well as those coerced by older adults often are ignorant of contraceptives usage and may not have the opportunity to practice safe sex.

#### Early sexual debut

Engaging in sex at an early age significantly contributes to unintended pregnancy and induced abortion among adolescents [39, 45, 47]. Similarly, in Ethiopia, sexual debut before the age of 18 was positively associated with unintended pregnancies [63]. Adolescents during this period of rapid physical development may be particularly vulnerable to exploitation by older individuals due to ignorance about their own physical and sexual development [17, 32, 33]. Encouraging adolescents to abstain from sexual activity during this critical period can help delay the onset of sexual activity and reduce the likelihood of unintended pregnancies. This underscores the importance of providing comprehensive sexual education and support services to adolescents to equip them with the knowledge and skills necessary to make informed decisions about their sexual health [45]. Additionally, instances of sexual activity resulting from rape or coercion contributed to the decision to undergo abortion [35]. This highlights the importance of addressing sexual violence, providing accessible healthcare services for survivors, and offering comprehensive sexual education to empower individuals to make informed decisions about their reproductive health.

#### Multiple sexual partners

Having multiple sexual partners is another individual factor associated with unintended pregnancies among adolescents. The review shows that adolescents who engage in sexual activity with multiple partners are more likely to experience unintended pregnancies [42, 43].

A qualitative study in Ghana identified multiple sexual partners as a factor contributing to unintended pregnancy among adolescents [64]. Alukagberie et al. [65] noted that financial constraints often precede engaging in multiple sexual partnerships, increasing the risk of adolescent pregnancy. Consequently, adolescents involved in multiple sexual partnerships may consider abortion due to heightened risks of unintended pregnancies and contraceptive failure [38], often influenced by emotional or social pressures. The accessibility of sexual and reproductive health services, which includes information about abortion, plays a significant role in shaping adolescents' decision-making processes [45]. Comprehensive sexual education programs should emphasize the importance of monogamous relationships and the risks associated with having multiple sexual partners. Additionally, efforts to increase access to contraceptives and reproductive health services can help empower adolescents to make safer choices regarding their sexual health [43, 56].

#### Protective factors

##### Contraceptive usage

Contraceptive usage is vital in preventing unintended pregnancies among adolescents [38, 41, 45, 47, 48, 55, 56]. It is one of the protective factors that can help to prevent adolescents’ unintended pregnancy and the risk of induced abortion. However, challenges such as limited access to contraceptives and stigma hinder their effective utilization [36, 50]. Marginalization of adolescents in accessing reproductive health services contributes to unintended pregnancy and abortion [66]. Cultural norms and restrictive laws exacerbate barriers to contraceptive access [67]. To address these challenges, comprehensive sexual education programs should not only educate adolescents about the importance of contraceptive use but also provide information on where and how to access contraceptive services [43, 50]. Community-based outreach initiatives can also play a significant role in increasing awareness and uptake of contraceptives among adolescents [68]. Addressing the barriers to contraceptive use in Nigeria can not only reduce unintended pregnancies but also potentially decrease the incidence of abortion among adolescents. Enhanced availability and promotion of contraceptives empower adolescents to make informed reproductive choices, potentially decreasing the need for abortion. This comprehensive approach to reproductive health can enhance overall adolescent well-being.

### Interpersonal level

#### Family dynamics

At the interpersonal level, family dynamics significantly impact adolescent reproductive health outcomes. The age and sex of the family head are crucial factors, with adolescent pregnancy being prevalent in households headed by males, possibly due to communication gaps regarding sensitive sexuality issues and insufficient warnings about associated dangers [51]. These findings underscore the importance of involving all family heads in reproductive health programs to enhance their understanding and support [27]. Miller et al. [69] found that parent–child closeness or connectedness, as well as parental supervision and regulation of adolescent activities, can decrease the likelihood of adolescent pregnancy. The study reflects that adolescents in polygamous households faced challenges in receiving individualized support and guidance regarding reproductive health and contraception, potentially leading to increased sexual activity [50, 52]. This highlights the importance of addressing family dynamics within polygamous households to promote adolescent sexual health. Additionally, the qualitative study from Ghana underscores the need to recognize polygamy as a contributing factor to unintended pregnancy among adolescents [64]. Efforts to mitigate these challenges could involve implementing family-based interventions aimed at improving communication and education on reproductive health within polygamous households.

Marital status also influences adolescents' access to reproductive health services, with married adolescents potentially facing fewer barriers compared to unmarried peers [40, 42, 46]. Addressing structural inequalities and discriminatory practices is crucial to ensure equitable access to reproductive health care for all adolescents, irrespective of marital status. Men's attitudes and behaviors significantly impact unmarried adolescents’ experiences of abortion, highlighting the importance of involving men in discussions and initiatives aimed at promoting reproductive health and rights [31, 47]. For instance, paternity doubt may create a lack of support and involvement from men [42, 56], as well as men's rejection of a pregnancy can lead to emotional distress and feelings of abandonment for women [31, 47]. Stigma and discrimination related to abortion can intensify the apprehension among unmarried adolescents about social and legal repercussions [31, 56, 57]. This complex situation further complicates their reproductive health decisions, potentially exacerbating their struggles in navigating the decision-making process regarding abortion.

### Organizational/community factors

Religious organizations often play a prominent role in shaping attitudes towards sexuality and reproductive health, which can impact adolescents' access to contraception and abortion services [39, 56]. At the organizational level, unemployment can create economic instability, limiting adolescents' access to resources and support for reproductive health care [48]. Place of residence, whether urban or rural, can affect access to healthcare services and information, potentially leading to disparities in reproductive health outcomes [26, 39, 51]. At the community level, factors such as place of residence, forced marriage, and ethnicity can also impact adolescent reproductive health outcomes. Many rural adolescents encountered restricted access to healthcare facilities and services, including contraception and abortion care, in contrast to their urban counterparts [26, 45]. Conservative social norms and economic barriers in rural communities increased vulnerability to unintended pregnancy [70]. Initiatives such as community-based education and awareness campaigns, along with comprehensive sexual education programs in rural schools, are essential to equip adolescents with the knowledge and skills needed to make informed decisions about their sexual health. Additionally, these programs serve to challenge conservative social norms and reduce stigma surrounding reproductive health issues.

Limited access to reproductive health services, coupled with cultural and religious stigma surrounding premarital sexual activity and abortion, leads to increased rates of unintended pregnancy and unsafe abortion among adolescents. Alukagberie et al. [65] similarly highlighted these issues. Addressing these issues requires a multi-faceted approach, including comprehensive sexual education, access to youth-friendly reproductive health services, advocacy for reproductive rights, and efforts to combat discrimination and stigma surrounding adolescent sexuality and abortion. Additionally, engaging religious and community leaders in promoting positive attitudes towards reproductive health and supporting adolescents' access to services is crucial for improving reproductive health outcomes among adolescents in Nigeria.

### Strengths and limitations

The study provides a thorough review of the prevalence and factors contributing to unintended pregnancy and abortions among unmarried adolescents in Nigeria, offering valuable insights into this complex issue. However, the review's reliance on studies published only in English could limit its scope by potentially excluding relevant literature in other languages. Additionally, the study did not delve into the methods used for abortions among adolescents, preventing the identification of specific abortion types performed. Despite these limitations, the review offers significant insights into the challenges and determinants of adolescent reproductive health outcomes in Nigeria, highlighting the need for further research and intervention. It's crucial to recognize that the findings may be specific to Nigeria and may not apply universally to other socio-cultural and healthcare contexts.

## Conclusions and recommendations

This review highlights the prevalence and diverse factors contributing to unintended pregnancy and abortions among unmarried adolescents in Nigeria, providing valuable insights into policy considerations. However, adolescents lack adequate SRH support and implementation, often resulting in discrimination against unmarried pregnant and adolescents. To address this issue, Nigeria must adopt a multisectoral approach to tackle the identified structural barriers, invest in evidence-based interventions, and prioritize the sexual and reproductive health of adolescents. Furthermore, addressing the shortage of literature on adolescent unintended pregnancy and abortion in Nigeria is imperative. Future research should focus on these phenomena and aim to strengthen comprehensive healthcare services for adolescent mothers. Nigeria can reduce unintended pregnancies and abortions among unmarried adolescents by enhancing research and healthcare services, empowering informed decision-making on reproductive health.

### Supplementary Information

Supplementary Material 1.

## Data Availability

Available from the corresponding author on reasonable request.

## Abbreviations

LMICs: Low and Middle-Income Countries
PRISMA: Preferred Reporting Items for Systematic Review and Meta- Analysis
SHB: Sexual Health Behaviour
SRH: Sexual and Reproductive Health
SES: Socio-economic status
