# Supplementary material for: Factors influencing unintended pregnancy and abortion among unmarried young people in Nigeria: a scoping review
Source: BMC Public Health. 2024 Jun 4;24:1494. doi: 10.1186/s12889-024-19005-8 (PMC11151533; doi:10.1186/s12889-024-19005-8)
Supplement: Supplementary file 1 — Supplementary Material 1. [file 12889_2024_19005_MOESM1_ESM.pdf]

Appendix 1: SEARCH RESULTS

Search number	Search Details	Results
**A**	**SCOPUS**	
#7	(((((#1) AND (#2)) AND (#3)) AND (#4)AND (#5)) AND (#6))	**3**
#6	Nigeria[Title/Abstract]	119,620
#5	((((((((((Adolescent[Title/Abstract]) OR (Adolescents[Title/Abstract])) OR (Adolescence[Title/Abstract])) OR (Teens[Title/Abstract])) OR (Teen[Title/Abstract])) OR (Teenagers[Title/Abstract])) OR (Teenager[Title/Abstract])) OR (Youth[Title/Abstract])) OR (Youths[Title/Abstract])) OR (Female Adolescent[Title/Abstract])) OR (Female	24345
#4	Unmarried[Title/Abstract]) OR (Single[Title/Abstract])	435
#3	Abortion[Title/Abstract]	307,899
#2	(Pregnancy[Title/Abstract]) OR (pregnancies[Title/Abstract])	234865
#1	((((Unintended[Title/Abstract]) OR (Unplanned[Title/Abstract])) OR (Unwanted[Title/Abstract])) OR (Mistimed[Title/Abstract])) OR (Accidental[Title/Abstract])	19,239
**B**	**PUBMED SEARCH**	
7	(((((#1) AND (#2)) AND (#3)) AND (#4)) AND (#5)) AND (#6)	**2**
6	Nigeria[Title/Abstract]	17,250
5	((((((((((Adolescent[Title/Abstract]) OR (Adolescents[Title/Abstract])) OR (Adolescence[Title/Abstract])) OR (Teens[Title/Abstract])) OR (Teen[Title/Abstract])) OR (Teenagers[Title/Abstract])) OR	167,692

	(Teenager[Title/Abstract])) OR (Youth[Title/Abstract])) OR (Youths[Title/Abstract])) OR (Female Adolescent[Title/Abstract])) OR (Female Adolescents[Title/Abstract])	
4	(Unmarried[Title/Abstract]) OR (Single[Title/Abstract])	833,180
3	Abortion[Title/Abstract]	13,886
2	(Pregnancy[Title/Abstract]) OR (pregnancies[Title/Abstract])	160,875
1	((((Unintended[Title/Abstract]) OR (Unplanned[Title/Abstract])) OR (Unwanted[Title/Abstract])) OR (Mistimed[Title/Abstract])) OR (Accidental[Title/Abstract])	36,763
**C**	**Web of Science**	
7	(((((#1) AND (#2)) AND (#3)) AND (#4)) AND (#5)) AND (#6)	**4**
6	TI:(Nigeria)	38616
5	(((((((((Adolescent) ORTI: (Adolescents)) OR TI: (Adolescence)) OR TI: (Teens)) OR TI: (Teen)) OR TI: (Teenagers)) OR TI: (Teenager)) OR TI: (Youth)) OR TI: (Youths)) OR TI: (Female Adolescent)) OR TI: (Female Adolescents)	496562
4	TI: (Unmarried) OR TI: (Single)	2936133
3	TI: (Abortion)	36703
2	((TI:(Pregnancy) OR TI: (pregnancies)	315201
1	((TI:(Unintended)) OR TI:(Unplanned)) OR TI: (Unwanted)) OR TI: (Mistimed)) OR TI: (Accidental)	111955
**D**	**SCIENCE DIRECT**	
	Nigeria AND ("Adolescent" OR "Adolescents" OR "Adolescence" OR "Teens" OR "Teen" OR "Teenagers" OR "Teenager" OR "Youth" OR "Youths" OR "Female Adolescent" OR "Female	**60**

	Adolescents") AND ("Unmarried OR "Single") AND ("Abortion) AND ("Pregnancy" OR "pregnancies") AND ("Unintended" OR "Unplanned " "Unwanted" OR " Mistimed OR "Accidental")	
**E**	**EBSCOHOST RESULTS**	
	Nigeria AND ("Adolescent" OR "Adolescents" OR "Adolescence" OR "Teens" OR "Teen" OR "Teenagers" OR "Teenager" OR "Youth" OR "Youths" OR "Female Adolescent" OR "Female Adolescents") AND ("Unmarried OR "Single") AND ("Abortion) AND ("Pregnancy" OR "pregnancies") AND ("Unintended" OR "Unplanned " "Unwanted" OR " Mistimed OR "Accidental")	**3**
**F**	**JSTOR**	
	Nigeria AND ("Adolescent" OR "Adolescents" OR "Adolescence" OR "Teens" OR "Teen" OR "Teenagers" OR "Teenager" OR "Youth" OR "Youths" OR "Female Adolescent" OR "Female Adolescents") AND ("Unmarried OR "Single") AND ("Abortion) AND ("Pregnancy" OR "pregnancies") AND ("Unintended" OR "Unplanned " "Unwanted" OR " Mistimed OR "Accidental")	**170**
**G**	**African Journals Online (AJOL)**	
	Nigeria AND ("Adolescent" OR "Adolescents" OR "Adolescence" OR "Teens" OR "Teen" OR "Teenagers" OR "Teenager" OR "Youth" OR "Youths" OR "Female Adolescent" OR "Female Adolescents") AND ("Unmarried OR "Single") AND ("Abortion) AND ("Pregnancy" OR "pregnancies") AND ("Unintended" OR "Unplanned " "Unwanted" OR " Mistimed OR "Accidental")	**311**
